# Clinical Evaluation of Single Versus Repeated Micro-Osteoperforations During Orthodontic Canine Retraction: A Randomized Clinical Trial

**DOI:** 10.7759/cureus.52026

**Published:** 2024-01-10

**Authors:** Bassem A Hashem, El-Hassanein H El-Hassanein, Ahmed A EL-Awady, Ahmed A Mohamed, Mohamed I Hashem, Majed M Alsarani, Farouk A Hussein

**Affiliations:** 1 Department of Orthodontics, Faculty of Dental Medicine for Boys, Al-Azhar University, Cairo, EGY; 2 Department of Orthodontics, Faculty of Dentistry, Menoufia University, Shebin El-Kom, EGY; 3 Department of Dental Health, College of Applied Medical Sciences, King Saud University, Riyadh, SAU

**Keywords:** 3d models, fixed orthodontic therapy, mini-screws, micro-osteoperforation, canine retraction

## Abstract

Introduction: In the majority of orthodontic premolar extraction cases, the canine retraction phase is the most laborious procedure. This randomized clinical trial aimed to assess the effect of single versus repeated micro-osteoperforations (MOPs) during orthodontic canine retraction.

Methods: In this split-mouth study, two equal groups of 18 patients who required maxillary first premolar extractions and fixed orthodontic therapy were randomly assigned (n=9). In Group I, MOPs were only performed once on one site before retraction, whereas in Group II, MOPs were performed on one site repeatedly once a month for four months. In both groups, the contralateral control sites received no MOPs. The canines were retracted using mini-screws and closed-coil nickel-titanium springs. Using the patients' 3D models, the primary outcome measure at four months was the amount of orthodontic canine distal movement. The amount of anchorage loss (AL), degree of molar rotation (MR) and canine rotation (CR), and degree of canine tipping (CT) were measured as the secondary outcomes. The comparison of mean changes in the primary and secondary outcomes between the groups was done using the independent sample t-test (p<0.05).

Results: The rate of canine retraction, degree of CT, and rotation were not significantly different between the two groups (p>0.05). Additionally, there were no statistically significant variations in the maxillary first MR and the degree of AL (p>0.05).

Conclusions: When maxillary canine retraction was performed with a single and repeated regimen of MOPs, comparable levels of distal CR and tipping were observed, along with an identical minimal degree of MR and AL.

## Introduction

Enhancing the dentofacial functioning and aesthetics of a patient is the main objective of orthodontic therapy. One of the primary issues with orthodontic therapy is the comparatively long duration of treatment, which can worsen the periodontal disease, demineralize enamel, resorb roots, and impair patient compliance [1]. Therefore, one of the main goals for all orthodontists is to minimize the duration of orthodontic therapy [2,3]. An average of 1.5 to 2 years is likely needed for conventional treatment with fixed orthodontic appliances. This time might vary depending on a number of factors, including the severity of the case, whether extraction or non-extraction therapy is required, whether orthognathic surgery is necessary, clinical experience, and patient compliance [4].

In the majority of orthodontic premolar extraction cases, the canine retraction phase is the most laborious procedure. Depending on the patient's age, conventional approaches yield a retraction rate ranging from 0.5 to 1 mm/month; hence, full canine retraction typically takes 5 to 6 months [4]. Many methods are currently used to minimize the duration of treatment and expedite orthodontic tooth movement (OTM). These methods are commonly designated to surgical and non-surgical techniques. Surgical techniques include osteotomies, corticotomies [5], and less invasive approaches like corticision, piezocision [6], and micro-osteoperforations (MOPs) [7]. Non-surgical techniques are resonance vibrators [8,9], laser irradiation [10], pulsed electromagnetic fields, and electrical currents, in addition to pharmacological approaches such as vitamin D and prostaglandin injection [11].

The purpose of MOPs, which are currently used to reduce the bone density surrounding teeth while preserving the bone density surrounding anchor teeth unchanged, is to create small, shallow, flapless osteoperforations between a tooth’s buccal or lingual roots on the surface of cortical plates. When the tooth is moved into an edentulous space, as in extraction cases, this treatment has been reported to be particularly beneficial [7,12]. MOPs have been suggested as a low-cost, safe, minimally invasive method that can be used to minimize treatment intervals and speed up OTM [7,13]. MOPs could be used at various treatment phases and in conjunction with orthodontic mechanics. Additionally, they were suggested to lessen the tension on anchor units and promote bone remodeling in regions with inadequate alveolar bone. Thus, in order to optimize the biological response to orthodontic stresses, MOPs can be repeated [14,15]. According to Alikhani et al., MOPs were able to increase the rate of OTM in their initial in vivo investigation [7].

Research on how MOPs affected orthodontic tooth movement revealed a range of results, from no effect at all to a 30% acceleration of tooth movement following one MOP in comparison to no intervention [7,16-20]. Additionally, previously available clinical studies utilized only one approach of a single application of MOPs for the acceleration of OTM.

Consequently, this study assessed the impact of MOPs' repeated approach on the rate of OTM and the impact of the frequent intervention on canine rotation (CR), tipping, and anchorage loss (AL), in addition to the molar rotation (MR). The rate of canine retraction from baseline to the fourth month was the primary outcome. The null hypothesis stated that there is no difference between the single and repeated application of MOPs and that MOPs do not expedite orthodontic treatment as compared to traditional orthodontic treatment.

## Materials and methods

Study design, sample, and eligibility criteria

A total of 18 patients were selected from the Orthodontic Department's outpatient clinic at Al-Azhar University to participate in this split-mouth randomized investigation. The Institutional Review Board of Al-Azhar University reviewed and approved the study protocol (Approval number 535/1147). The study is also registered on ClinicalTrials.gov (ID: NCT04868721). All participants in the study, including patients and their parents, filled out an informed consent to allow the usage of the data for research. The following parameters were used to calculate the sample size using the G*power (v.3.1) statistical program: 80% power, two-tailed significance level α=0.05, and considering clinically relevant variations in tooth movement [20]. Nine patients in each group (n=9 per group), or a total of 18 patients, were the estimated lowest sample required to have adequate power to determine a clinical difference.

Male and female patients who fulfilled the following criteria were deemed eligible for the study: patients aged 15-22 years with complete permanent dentition and malocclusion that required the extraction of the maxillary first premolars and the retraction of the canines, and they had to have good oral hygiene and periodontal health. This study did not include patients with compromised periodontium, craniofacial anomalies, trauma history, bruxism, parafunctional behaviors, prior orthodontic treatment, long-term medications that might interact with OTM, or poor oral hygiene [7,21]. The following factors were used to determine whether a patient should stop receiving treatment: uncooperative patients, frequently broken appliances, repeated missed appointments, and inability to maintain good oral hygiene through the treatment.

Randomization and group allocation

According to the MOP application, each patient was randomly allocated into two groups for the split-mouth clinical trial. In Group I, MOPs were carried out once only on one site before retraction, whereas in Group II, MOPs were repeatedly carried out on one site. The contralateral control site involved the retraction of canines without the use of MOPs. Every MOP protocol was assigned at random to the left or right site. Coin tosses were used for the randomization and group allocation processes to prevent selection bias.

Blinding

Blinding the patient or the operator was not possible. However, blinding was maintained at the analysis stage. All 3D digital models were coded to conceal the investigator's knowledge of the locations where the MOPs were used.

Interventions

Every patient who was enrolled in the study had their diagnosis established through a comprehensive clinical examination, routine orthodontic records, and an evaluation of study models, photos, and cephalometric and panoramic radiographs.

Orthodontic appliance

Every patient received conventional orthodontic therapy with fixed appliances. Light-cured orthodontic adhesive composite (Ormco Corp, Glendora, CA, USA) was used to directly bond pre-adjusted metallic brackets with a 0.022-inch slot (Damon Q, Ormco Corporation, Orange, CA, USA) from the right to the left maxillary second premolars, except for the maxillary first premolars. In addition, the maxillary first molars were also directly bonded using single buccal molar tubes with a 0.022-inch slot and hooks (American Orthodontics, USA).

Since extraction is regarded as a surgical insult that may increase inflammatory markers, maxillary first premolar extractions were performed four months before the application of MOPs. The patients were referred to the oral surgery department for the extraction of upper first premolars (minimal traumatic extraction) before the orthodontic bonding. Four months was considered adequate for the extraction socket to heal fully, as it has been claimed that 85% of mature lamellar bone will replace the woven bone during the healing process [22,23]. Leveling and alignment were carried out in both arches using a sequence of round NiTi archwires (Super Elastic Nitanitum Archwires, Ortho Organizer, USA), 0.014, 0.016, and 0.018-inch wires. This was followed by the insertion of rectangular wires (0.016×0.022-inch) untill reaching a final working rectangular 0.016×0.022-inch straight archwire (Ortho Organizer, Carlsbad, CA, USA) for three weeks before retraction of maxillary canine to ensure passive sliding of the archwire through the bracket slots [21,22,24,25].

Anchorage preparation

To reduce the unintended movement of the posterior teeth during canine retraction, mini-screws were used. The mini-screws (1.6 × 8 mm, AbsoAnchor Microimplant, Korea) were placed between the upper first molar and second premolar at the level of the mucogingival junction with the intent to be used as both direct and indirect anchorage following initial leveling and alignment [17,18,21,26].

Canine retraction phase

Prior to the canine retraction phase, the upper incisors were ligated with a figure of eight 0.009-inch wire to stabilize the anterior segment [21,24,27]. The maxillary second premolars were ligated to the first maxillary molars with a 0.009-inch wire on each site [22,28]. On 0.016×0.022-inch straight archwires, the maxillary canine retraction was started. Following the experimental site's complete leveling, alignment, and MOP application, the retraction was initiated simultaneously on both sites.

A Niti closed coil spring (American Orthodontics, Washington Avenue, USA) was used to retract canines. It was attached between the mini-screw head and the hook of the maxillary canine bracket [14,21]. A 0.009-inch ligature wire that was fastened to the canine bracket's distal wing held the spring in situ. A 0.009-inch ligature wire was used to ligate the canine bracket to the archwire. The force of the NiTi closed coil spring was measured using a tension gauge (Correx tension gauge, Dentaurum) with the ends of the spring attached to the mini-screw head and the gauge's measuring end to determine the appropriate length to produce a standardized force of 150 gm. The force was measured at the beginning of the canine retraction and repeated at every subsequent appointment (every month) to adjust the proper length to produce a standardized force for retraction [21,24,29-31].

Clinical MOP procedure

Once the canines were prepared for retraction, MOPs were performed [16,17,19,22]. The patients were instructed to clean their mouths for a minute with chlorhexidine antiseptic mouthwash. The next step was to administer local anesthesia (2% lidocaine with 1:100,000 epinephrine). One researcher (B.A.) performed the MOP procedure on the maxillary left or right site based on randomization.

Mini-screws measuring 1.6 mm in diameter and 8 mm in length were used at three places distal to the canine to perform three MOPs, each measuring 1.6 mm in width and 4 mm deep into the bone. To ensure that the procedure was standardized, a set of three perforations spaced equally from one another and halfway through the extraction space were used as bleeding sites to pinpoint the exact location of screw insertion. The three points of insertion were spaced equally between the canine and the second premolar: the first point was 6 mm from the free gingival border, the second point was 5 mm from the first point, and the third point was 5 mm from the second point. After inserting the mini-screw 4 mm into the bone, it was removed. Endodontic files and a rubber stopper were used to standardize the depth of perforation.

Outcomes

**Figure 1:**
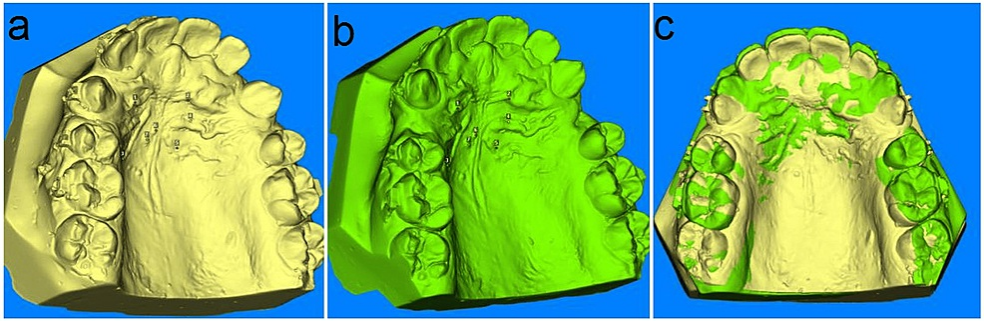
Registration of reference points in the digital models at the rugae area at T0 (a), registration of reference points in the digital models at the rugae area at T4 (b), and superimposed digital models (c)

The key outcome measure at four months was the amount of distal movement of orthodontic canines. Alginate impressions were obtained before canine retraction and after four months, and study models were produced. To generate 3D digital models, the study models were scanned with a Primescan scanner (Sirona Primescan Scanner, Dentsply Sirona, USA) with a precision of 0.0021 mm. The scanned maxillary models were then imported to Dolphin software (Dolphin Imaging version 11.9, Patterson Inc., Chatsworth, California, USA), where they were virtually positioned in a mutual position similar to the baseline scanned model, superimposing the baseline 3D digital model (T0) on the 4th month's (3D) digital model (Figure 1) [32,33].

**Figure 2:**
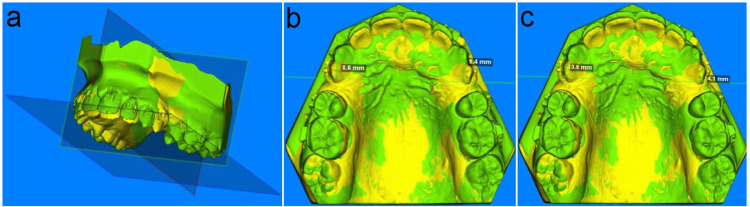
Reference planes for the measurements (a), measurements of 3D models between cusp tips of maxillary canines and rugae line at T0 (b), and measurements of 3D models between cusp tips of maxillary canines and rugae line at T4 (c)

The magnitude of canine distal movement was measured in the MOPs and control sites in both groups and recorded in mm. Following superimposition, the distance difference between the maxillary canine cusp tip and the rugae line perpendicular at T0 and T4 was measured (Figures 2b, 2c) [18,33-35].

**Figure 3:**
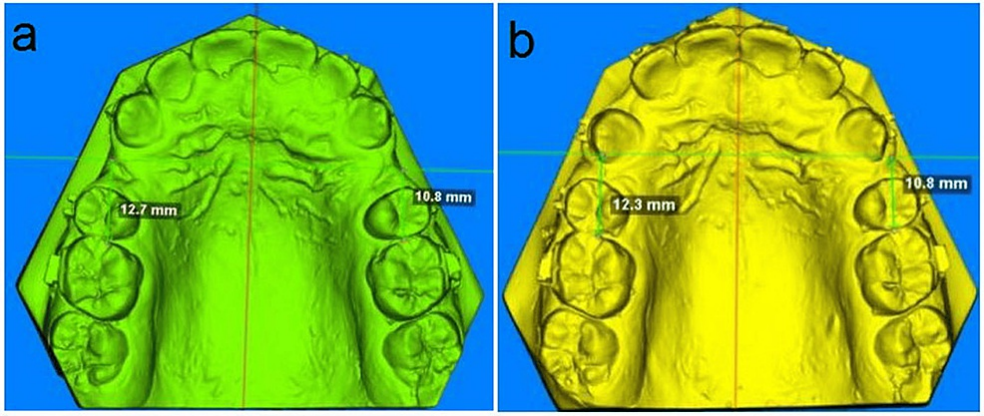
Measurements of 3D models between the mesial surface of maxillary first molar and rugae line at T0 (a) and measurements of 3D models between the mesial surface of maxillary first molar and rugae line at T4 (b).

The most medial end of the third rugae area, which was perpendicular to the mid-palatal raphe line at T0, defined the rugae line [36,37]. The secondary outcome included measuring the degree of canine tipping (CT), the degree of MR and CR, and the amount of AL. The difference in length between the most conspicuous location on the maxillary first molar's mesial surface and the rugae line at T0 and T4 of the superimposed 3D models was used to calculate the amount of AL for the scanned models. The result was expressed in mm (Figures 3a, 3b) [21,38]. The distal angle difference between the canine's longitudinal axis and the occlusal plane at T0 and T4 of the superimposed 3D models was used to assess and record the degree of maxillary CT from the buccal viewpoint [39,40].

The difference in the internal angle between the mid-palatal raphe line and the line that passes through the mesial and distal contact point of the maxillary canine at T0 and T4 of the superimposed 3D models was used to measure and record the degree of maxillary CR from the occlusal view. The result was expressed in degrees [6,25,35,37,38,41]. The difference in internal angle between the mid-palatal raphae and the line that passes through the tips of the mesiopalatal and distobuccal cusps of the maxillary first molar at T0 and T4 of the superimposed 3D models was used to measure and record the degree of rotation of the first molar [42].

Statistical analysis

The data were statistically analyzed using IBM SPSS Statistics for Windows, Version 25 (Released 2017; IBM Corp., Armonk, New York, United States). The Shapiro-Wilk normality test demonstrated that the data had a normal distribution. As a result, parametric tests were used for the statistical analysis. The mean and SD were used to express the quantitative variables. Descriptive statistics, including mean differences, SD, standard errors, and percentage changes in all measurements, were computed for each variable in both groups. A t-test for independent samples was employed to compare the mean differences between the two groups. With 95% confidence intervals, significance was established at the 0.05 confidence level. Tests for intra-observer reliability included the paired t-test and the interclass correlation coefficient test.

## Results

**Figure 4:**
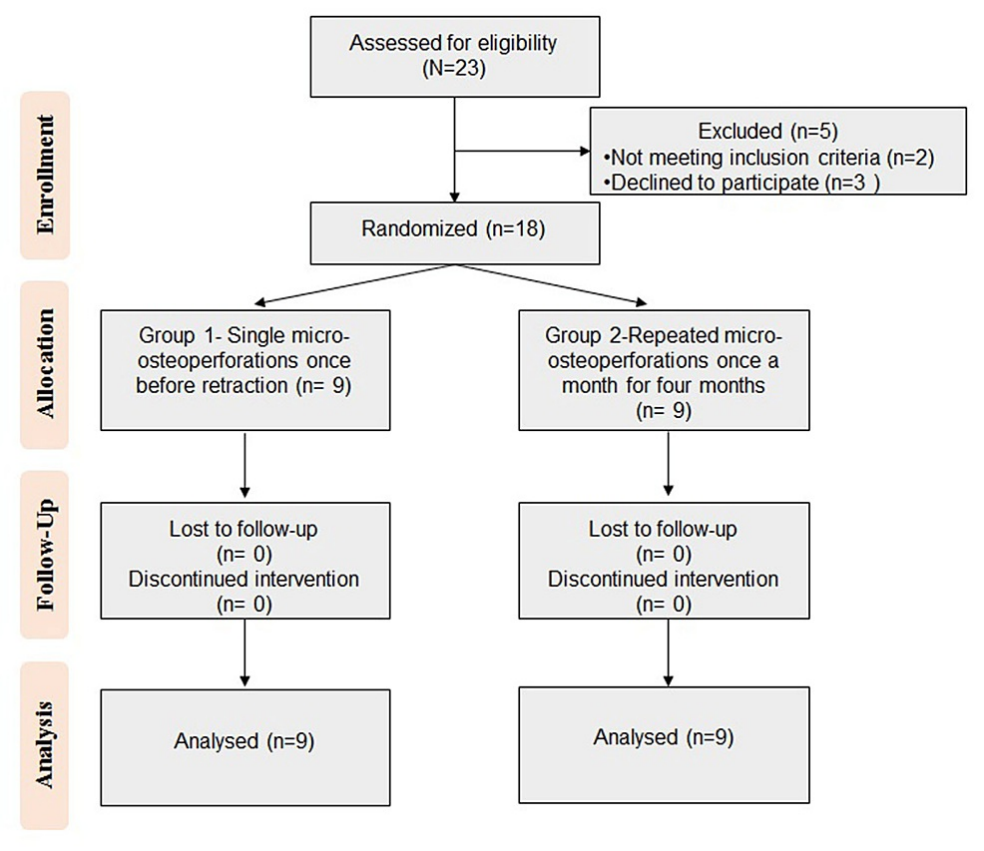
CONSORT flow diagram outlining the study process

The clinical trial's flow chart, which outlines the investigation process, is shown in Figure 4. A total of 23 patients were assessed for eligibility, two patients did not meet the inclusion criteria and three patients declined to participate. The remaining 18 patients were allocated into two groups according to the treatment received (n=9). All 18 patients completed the trial and were available for final analysis. 

Intra-observer reliability measurements

Six randomly chosen cases, or 33% of the sample, had all clinical assessments for the above parameters revised four weeks later by a single examiner blind to the nature of the study groups. The reliability coefficient of 0.973 to 0.987 demonstrated good agreement. Furthermore, no significant difference (p>0.05) was found when the mean difference between the first and second measures was examined.

Primary outcomes

Between the experimental sites in both groups and between the control and experimental sites within each group, there was no significant difference in orthodontic canine distal movement (p>0.05) (Table 1).

**Table 1:** Descriptive data and comparison of the mean changes of the measured parameters between the groups at the control and experimental sites at four months CD: Canine Distalization, CT: Canine Tipping, CR: Canine Rotation, AL: Anchorage Loss, MR: Molar Rotation, (o): Degrees, P: Probability Level, SD: Standard Deviation, SE: Standard Errors, Sig: Significance, NS: Non-significant •Paired t-test.

	Parameters	Groups	Control group			Experimental group			Difference		T-test•	p-value	Sig.
			Mean	SD	SE	Mean	SD	SE	Mean	SE			
Maxillary Canine	CD (mm)	Group I	3.644	1.158	0.386	3.700	0.949	0.316	0.056	1.022	0.163	0.875	NS
		Group II	3.989	1.056	0.352	4.411	1.505	0.502	0.422	0.884	1.432	0.190	NS
	CT (^o^)	Group I	5.989	3.783	1.261	4.089	3.558	1.186	-1.900	3.486	-1.635	0.141	NS
		Group II	7.456	1.557	0.519	5.744	3.784	1.261	-1.711	3.921	-1.309	0.227	NS
	CR (^o^)	Group I	10.467	3.163	1.054	11.744	2.290	0.763	1.278	3.387	1.132	0.290	NS
		Group II	11.478	3.478	1.159	12.456	5.000	1.667	0.978	5.894	0.498	0.632	NS
Maxillary 1^st^ molar	AL (^o^)	Group I	0.767	0.530	0.178	0.556	0.590	0.197	-0.211	0.408	-1.554	0.159	NS
		Group II	0.300	0.364	0.121	0.322	0.286	0.095	0.022	0.514	0.130	0.900	NS
	MR (^o^)	Group I	1.667	0.709	0.236	1.400	0.669	0.223	-0.267	0.566	-1.414	0.195	NS
		Group II	2.144	1.665	0.555	1.644	0.886	0.295	-0.500	1.849	-0.811	0.441	NS

Secondary outcomes

Orthodontic canine distal movement did not significantly differ between experimental and control sites within each group nor between experimental sites in the two groups (p>0.05) (Table 2).

**Table 2:** Descriptive data and comparison of the mean changes of the measured parameters between the groups at the control and experimental sites of the groups CD: Canine Distalization, CT: Canine Tipping, CR: Canine Rotation, AL: Anchorage Loss, MR: Molar Rotation, (o): Degrees, P: Probability level, SD: Standard Deviation, SE: Standard Errors, Sig: Significance, NS: Non-significant •Paired t-test.

	Parameters	Site	Group I				Group II			Difference		T-test•	p-value	Sig
			Mean	SD	SE		Mean	SD	SE	Mean	SE			
Maxillary Canine	CD (mm)	Control	3.644	1.158	0.386	3.989		1.056	0.352	-0.711	0.593	-1.199	0.248	NS
		Experimental	3.700	0.949	0.316	4.411		1.505	0.502	-0.344	0.522	-0.659	0.519	NS
	CT (^o^)	Control	5.989	3.783	1.261	7.456		1.557	0.519	-1.656	1.731	-0.956	0.353	NS
		Experimental	4.089	3.558	1.186	5.744		3.784	1.261	-1.467	1.364	-1.076	0.298	NS
	CR (^o^)	Control	10.467	3.163	1.054	11.478		3.478	1.159	-0.711	1.833	-0.388	0.703	NS
		Experimental	11.744	2.290	0.763	12.456		5.000	1.667	-1.011	1.567	-0.645	0.528	NS
Maxillary 1^st^ molar	AL (^o^)	Control	0.767	0.534	0.178	0.300		0.364	0.121	0.233	0.219	1.068	0.301	NS
		Experimental	0.556	0.590	0.197	0.322		0.286	0.095	0.467	0.215	2.167	0.051	NS
	MR (^o^)	Control	1.667	0.709	0.236	2.144		1.665	0.555	-0.244	0.370	-0.660	0.518	NS
		Experimental	1.400	0.669	0.223	1.644		0.886	0.295	-0.478	0.603	-0.792	0.440	NS

## Discussion

One primary concern for the majority of orthodontic patients, especially adults, is the duration of orthodontic treatment. According to the study by Mavreas and Athanasiou, comprehensive orthodontic therapy typically takes 1.5 - 2 years to complete, which may increase the likelihood and severity of any side effects [3]. The incidence of caries, periodontal issues, white spot lesions, and orthodontically induced root resorption is directly correlated with treatment duration [1]. The purpose of the current study was to compare two approaches of MOPs as a less invasive surgical technique during orthodontic canine retraction. In Group I, MOPs were performed only once on one site prior to retraction, and in Group II, MOPs were performed in a repeated manner on one site only every month for four months. The repeated MOPs were supported by the hypotheses that increasing the amount of surgical trauma will increase osteoclastic activity [43] and that increasing the amount of biological procedure could reinduce the regional acceleratory phenomenon (RAP) and the acceleration of OTM [44].

This study's primary outcome was to evaluate the rate and quantity of maxillary canine distal movement. The secondary outcome was the assessment of maxillary CR and CT, the mesial movement (AL), and the rotation of the maxillary first molar. According to Feizbakhsh et al., age affects the rate at which teeth move because of bone density variations and osteoclasts' production and activation [20]. In order to mitigate the impact of age on the current investigation, the sample mean age was 16.78 ± 2.22 years for Group I and 17.56 ± 2.65 years for Group II. During the trial, homogeneity across the tested groups was achieved without any inherent bias by applying an independent t-test to verify the baseline measurements of age, pre-treatment inconsistency, and pre-retraction extraction space, which were matched as much as possible.

The bias between the maxilla and mandible's bone density was eliminated in this study by evaluating only the maxillary arch [15,17,18,33]. The right and left sites were randomly assigned to either group to eliminate the effects of uneven occlusion forces that may arise from habitually chewing on one side and to minimize left-right bias [45]. NiTi closed coil springs were used to retract the maxillary canines because they could provide a consistent light force of 150 g for the duration of the therapy. Furthermore, they do not exhibit early force degradation like elastomeric chains or elastic modules and might promote better dental cleanliness. A force gauge was also utilized to ensure that the retraction force at both sites began at the same level [6,17,22].

The force applied to the maxillary canines during canine retraction from the mini-screws was used in the current study as a direct anchorage method. The indirect anchorage was also used by passively ligating the first molar, second premolar, and maxillary second premolars to mini-screws to potentially stop the posterior teeth from moving mesially [17,21,24]. These measures were undertaken to allow accurate assessment of pure distal canine movement as much as possible.

The timing of tooth extractions can affect the amount of OTM by triggering inflammatory responses that may make it more difficult to assess the impact of MOPs [20]. It was demonstrated in an earlier study that the RAP lasts for about four months following the stimulation [46]. To achieve the previously recommended pure biological reaction of MOPs without the interference of extraction-induced RAP, all current participants underwent extractions at least four months before the intervention. Four months was considered adequate for the extraction socket to heal fully, as it has been claimed that 85% of mature lamellar bone will replace the woven bone during the healing process [21-23].

The techniques utilized to assess the canine distal movement varied significantly among the investigations. The orthodontic canine retraction was determined using the following methods in previous studies: the distance between the lateral incisor and canine [7], variations in the distal surface of canine position [17], the distance between the distal surface of canine and the mesial surface of the premolar [47], the distance between the canine and mini-screw [20], the distance of canine cusp to the mid-incisal surface of lateral incisor [48], and the distance between the canine tip and mesiobuccal cusp tip of maxillary 1st molar [49]. Furthermore, Alikhani et al. [7] and Sivarajan et al. [49] used study casts and intraoral measurements to measure canine movement, while Alkebsi et al. [17], Feizbakhsh et al. [20], Attri et al. [47], and Kundi et al. [48] used 3D models.

Digital assessments of the maxillary canine retraction, rotation, tipping, mesial movement (AL), and rotation of the maxillary first molar were performed using three-dimensional scanned models per other research studies [18,33-35]. However, other authors used conventional model analysis to assess the distance of orthodontic canine retraction, which might be influenced by factors other than the distance moved by the maxillary canine, such as anchorage loss, molar, and canine rotations [47]. In the current study, the rate achieved by the maxillary canine in Group I (single MOPs) measured by the superimposition of 3D digital models was found to be 3.70 mm ± 0.95 mm, 3.64 mm ± 1.16 mm for experimental and control sites, respectively after four months. Regarding Group II (repeated MOPs), the rate of maxillary canine was 4.41 mm ± 1.51 mm and 3.99 mm ± 1.06 mm for the experimental and control sites, respectively, throughout the four-month observation period. After four months, there was no discernible variation in the maxillary canine's total distance moved when the rate of canine retraction was compared between the two groups. This outcome is in agreement with previous studies by El-Awady et al. [50], Alkebsi et al. [17], Alqadasi et al. [18], Aboalnaga et al. [16] and Li et al. [51] for the single approach of MOPs. Also, Bolat et al. [52] and Fattori et al. [53] revealed similar outcomes in the context of repeated MOPs.

Moreover, following a four-month observation period, the AL measurements from the 3D digital models were minimal: 0.56 ± 0.59 mm and 0.77 ± 0.53 mm for experimental and control sites in Group I, respectively, and 0.32 ± 0.29 mm and 0.30 ± 0.36 mm for experimental and control sites in Group II, respectively. There was no significant variation between these minimal values in both groups throughout the observation period. These results are in line with those of Alkebsi et al. [17], Alqadasi et al. [18], Aboalnaga et al. [16], Raghav et al. [54], and El-Awady et al. [50] for a single MOP protocol, and Jaiswal et al. [33] for the repeated one. Interestingly, a recent systematic review concluded that MOPs do not have any significant effect on anchorage loss [55]. The minimal anchorage loss observed in this study could be anticipated due to the previously mentioned measures that were utilized to control the anchorage as much as possible.

The present degrees of maxillary canine tipping were 4.09 ± 3.56 and 5.99 ± 3.78 for the experimental and control sites in Group I, respectively, and 5.74 ± 3.78, and 7.46 ± 1.56 for the experimental and control sites in Group II, respectively. The maxillary canine rotations were 11.74 ± 2.29 and 10.47 ± 3.16 for the experimental and control sites in Group I, respectively, and 12.46 ± 5.01 and 11.48 ± 3.48 for the experimental and control sites in Group II, respectively. Also, the degree of maxillary 1st molar rotation was 1.40 ± 0.67 and 1.67 ± 0.71 for the experimental and control sites in Group I, respectively, and 1.64 ± 0.89, and 2.14 ± 1.67 for the experimental and control sites in Group II, respectively. These aforementioned findings showed no significant variations between the groups throughout the follow-up period. These outcomes are in agreement with the results of Alkebsi et al. [17], Alqadasi et al. [18] for a single MOP approach, Jaiswal et al. [33], and Haliloglu-Ozkan et al. [15] for repeated MOP approaches.

Based on the current results, MOPs may accelerate orthodontic tooth movement. However, this impact is clinically negligible and does not last throughout the treatment. It is crucial to recollect that the potential for a cross-over effect from the experimental site to the control site is the primary drawback of the split-mouth design. It was thought that even if the cross-over did occur, its effect would only be observed during the initial exposure period. Therefore, the control sites for both groups were compared to each other in the current study to exclude the possibility of a cross-over effect. The findings revealed insignificant variations between the control groups of both approaches. Consequently, it could be considered that there was no cross-over effect from either MOP approach to the control sites in this study.

One of the current study's drawbacks may be that blinding the patient and operator was only possible during the analysis stage. A bigger sample size, the type of malocclusion being treated, and a more extended evaluation period could all impact the study outcomes. Future research could examine how these techniques affect treatment’s long-term stability, root resorption, and tooth vitality.

## Conclusions

Based on the outcome and within the limitations of the study, micro-osteoperforations may accelerate orthodontic tooth movement. However, this impact is clinically negligible and does not last throughout the duration of the treatment. Maxillary canine retraction with a single and repeated protocol of MOPs demonstrated similar degrees of distal canine tipping and rotation. Both protocols of MOPs showed an equivalent minimal extent of anchorage loss and molar rotation during retraction of maxillary canine.
